# A new cultivation method for microbial oil production: cell pelletization and lipid accumulation by *Mucor circinelloides*

**DOI:** 10.1186/1754-6834-4-15

**Published:** 2011-06-02

**Authors:** Chunjie Xia, Jianguo Zhang, Weidong Zhang, Bo Hu

**Affiliations:** 1 State Key Laboratory of Chemical Resource Engineering, Beijing University of Chemical Technology, Beijing, China; 2 Department of Bioproducts and Biosystems Engineering, University of Minnesota, MN, USA; 3 Department of Bioproducts and Biosystems Engineering, University of Minnesota, 316 BAE, 1390 Eckles Avenue, St. Paul, MN 55108-6005, USA

## Abstract

The recent energy crisis has triggered significant attention on the microbial synthesis of lipids, which comprise the raw material for biodiesel production. Microbial oil accumulation with filamentous fungi has great potential because filamentous fungi can form pellets during cell growth, and these pellets are much easier to harvest from cell broth. This paper focuses on the cell pelletization process of the oleaginous *Mucor circinelloides*. We have studied the effect of various cultural conditions on pelletized cell growth and lipid accumulation. This study is the first to report that pH adjustment during cell growth plays a key role in pellet formation of *M. circinelloides *and describes a handy method by which to induce cell pelletization in submerged fungal cultivation. Our study reveals that cell growth and lipid production are not significantly affected by pelletization and that lipid accumulation is triggered at stressed conditions, such as a high carbon-to-nitrogen ratio and high temperature.

## Background

Biomass-based biofuel production has emerged as a major approach to enabling energy independence, reducing greenhouse gas emissions, revitalizing rural communities and enhancing sustainable economic development. The accumulation of lipids, which comprise the raw material for biodiesel production through transesterification reactions, has been receiving a tremendous amount of attention recently, especially with regard to microalgae because of its high content of oil accumulated in certain stressed cultural conditions [1,2]. In addition to oil-producing microalgae, many species of yeast and filamentous fungi have the capability to synthesize lipids in their cells. Numerous studies have revealed the possibility of significantly accumulating lipids through the use of many oleaginous yeasts on different substrates, such as industrial glycerol, sewage sludge, whey permeate, sugar cane molasses and rice straw hydrolysate [3-9]. However, these strains are usually sensitive to the common inhibitors generated during lignocellulosic hydrolysis, and certain detoxification steps are needed prior to their fermentation [9-11]. By utilizing glycerol, acetic acid, soluble starch, wheat straw, wheat bran and so forth, some oleaginous filamentous fungi can be used to produce lipids [12-15]. The capabilities of these oleaginous fungi provide their potential to utilize sugars in pretreated lignocellulosic hydrolysate. The fatty acid profile of the microbial lipids is quite similar to that of conventional vegetable oils. Therefore, oleaginous filamentous fungi are suggested as a favorable feedstock for a sustainable biodiesel industry [14,16].

The harvest of fungal cells can be easier than microalgae and yeast cells because of their filamentous growth. In submerged cultures, many filamentous microorganisms tend to aggregate and grow as pellets or granules. Pelletized fungal cells can potentially perform high-density cultivation with significantly higher productivity [17]. Also, fungal pellets can be easily separated from the broth by using a simple filtration method. The latter feature especially aroused interest because of possible applications in lipid accumulation to generate biofuel, considering the economically infeasible separation costs of current microbial biodiesel processes. Although there are several techniques under development, the most commonly used harvest methods for the oleaginous cells are still through centrifugation-related techniques. The high costs of these methods have been the major obstacle to using the algae-to-fuel or yeast-to-fuel approach [18]. There have not been any comprehensive studies on the use of pelletized fungal conversion for microbial biodiesel production, although it was reported that pellet formation might facilitate γ-linolenic acid production [19,20]. Therefore, the present research was focused on an oleaginous filamentous fungus to study its cell pelletization and oil accumulation so that we can provide an alternative method for microbial biodiesel production featuring easy cell harvest. The filamentous fungus *Mucor circinelloides *was chosen as the model microorganism to study this new cultivation technique because *M. circinelloides *has been widely researched for its lipid production, and one of these fungus strains, CBS277.49, has been selected by the Department of Energy as a potential lipid producer to sequence its whole genome; in addition, the transformation process of its mycelium into biodiesel has been investigated by several researchers [21-24].

## Materials and methods

### Fungal strain and inoculums preparation

*M. circinelloides *(ATCC1216B; American Type Culture Collection, Manassas, VA, USA) was selected as our model organism for this investigation. A spore suspension was used for inoculation of the flask cultures. To obtain spores, agar plates with the sporulation medium (24 g L^-1 ^potato dextrose broth with 20 g L^-1 ^agar) were plated out with spores from a frozen stock (stored in 25% glycerin at -70°C) and incubated for 6 days at 27°C. After growth, 10 mL of sterilized water were added into the agar plates to release the aerial mycelium. The number of spores in the suspension was counted by using an optical microscope (National Optical & Scientific Instruments Inc., San Antonio, TX, USA).

### Culture medium

The flask cultural medium contained glucose (20 g/L) as the carbon source, both yeast extract (1 g/L) (Acros Organics #AC611801000; Fisher Scientific) and NH_4_Cl (1.5 g/L) as the nitrogen source, KH_2_PO_4 _(6 g/L) and MgSO_4 _7 H_2_O (1.2 g/L). The culture medium may change as specified in each cultural condition, together with other important growth factors, such as initial pH level, culture temperature and so on.

### Cultivation methods

Flask cultures of *M. circinelloides *were carried out in 250-mL Erlenmeyer flasks containing 100 mL of medium on a rotary shaker (Innova 42R; New Brunswick Scientific, Edison, NJ, USA) at 180 rpm for 6 days. The culture medium was always sterilized before fungal spores were introduced for inoculation. Three fermentation runs per culture experiment were performed. Unless specifically addressed, the initial pH of the culture medium was 3.0 before sterilization and was measured and adjusted to 5.30 after 18 hours in culture to induce fast cell growth and cell pelletization. The cultivation temperature was 27°C, and the inoculum size was 1.16E4/L fungal spores. The cultivation conditions were the same for all experiments unless specifically indicated otherwise.

### Analytical methods

Glucose concentration was estimated by using a dinitrosalicylic acid assay [25]. A Canon PowerShot SD1200 IS photograph (Canon, Japan) was used to observe the pellet morphology. All the pellets from each flask were poured into one Petri dish with the label on the left side and the ruler on the top to take the photograph. If the pellet number was too high and a single Petri dish could not display all of them, then pellets were split into two or more Petri dishes to allow clear visualization. The size of the pellets was measured using Image-Pro Plus 6.0 software (Media Cybernetics Inc.). The concentration of ammonia was measured using the phenate method [26]. The mycelia fungi were separated by centrifugation (9,000 rpm for 5 minutes), and harvested fungi were washed twice with distilled water, frozen overnight at -70°C and then freeze-dried to a constant weight by using the Virtis FreezeMobile 25EL freeze dryer (USA). Before the extraction of lipids, the biomass was pulverized. The lipids were extracted from the dried biomass by using a chloroform, methanol and water solution. All values derived are the means of triplicate measurements.

## Results

### Growth curve of *M. circinelloides*

**Figure 1:**
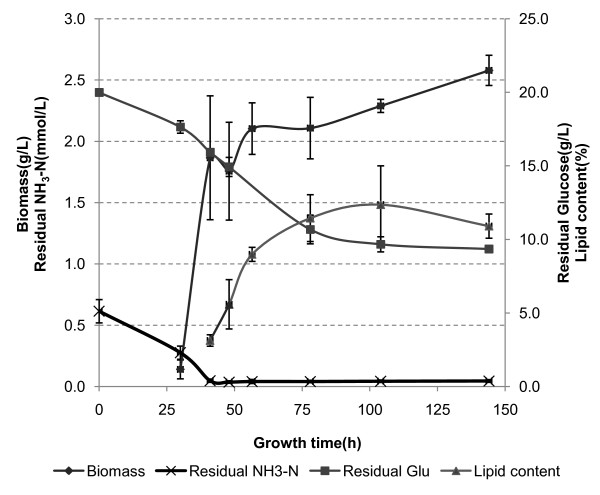
**Growth curve of *Mucor circinelloides *(24°C, nitrogen:yeast extract 1 g/L)**.

*M. circinelloides *has the key characteristics of a typical oleaginous species as shown in Figure 1. The biomass concentration drastically increased after the lag phase, became almost stagnant after 41 hours of cultivation and then started to decline after 144 hours. The nitrogen level in the fermentation broth was quickly depleted as the ammonia concentration dropped to around zero after 41 hours. Then the lipid content of the fungal cells gradually increased from 2% to over 12%. Glucose was consumed during the cell growth and lipid accumulation, but nearly 50% of the initial glucose remained unutilized in the fermentation broth. It has been reported that *M. circinelloides *usually synthesizes the lipid within 24 hours after nitrogen depletion [21], and our results seem to confirm this conclusion. The final lipid content is not very exceptional, being significantly lower than that of many other oleaginous fungal species that are currently used in active research [15,27].

### Initial pH effects on the cultivation of *M. circinelloides*

Culture pH significantly influenced the cell growth of *M. circinelloides*. A pH drop was recorded during the early stage of cell cultivation, probably due to some unknown acid excretion, causing slow cell growth in the later stage. CaCO_3 _power (0.4 g/100 mL) was added at 18 hours of cell culture as the recommended method to induce cell pelletization, with another effect of increasing the pH of the fermentation broth [17]. As shown in Table 1 the final cell biomass concentrations were relatively the same when the initial pH changed from 3 to 9, with a slight trend to peak between 6 and 7, while it significantly dropped when the initial pH was over 10. Cell growth was totally inhibited when the initial pH was 1 and 2 (data not shown), and even with the addition of CaCO_3 _to increase pH at 18 hours of culture, there was still no measurable amount of biomass generated. The lipid content of cultures with an initial pH of 3 was slightly higher than that of the remaining cultures, which might imply that pH is a stressing factor triggering lipid accumulation. Only the cultures with an initial pH of 3 showed obvious formation of fungal pellets; therefore, an initial pH of 3 was chosen as one of the cultivation conditions in the subsequent experiments conducted to study fungal pellet formation.

**Table 1:** Effect of initial pH on growth and lipid content of *Mucor circinelloides*^a^

	Initial pH							
	
Parameter	3.0	4.0	5.0	6.0	7.0	8.0	9.0	10.0
Mean biomass concentration, g/L (± SD)	1.2466 ± 0.058	1.3137 ± 0.043	1.3508 ± 0.022	1.3751 ± 0.044	1.3675 ± 0.036	1.3033 ± 0.021	1.2400 ± 0.200	0.7953 ± 0.345
Mean lipid content, % (± SD)	30.87 ± 15.72	16.78 ± 9.83	17.71 ± 1.29	14.46 ± 4.42	12.93 ± 5.03	10.97 ± 2.60	14.28 ± 9.47	12.38 ± 7.24
Pellet formation	○	×	×	×	×	×	×	×

^a ^SD: standard deviation; ○: pellets; ×: no pellets.

### CaCO_3 _addition effects on pelletization of *M. circinelloides*

**Figure 2:**
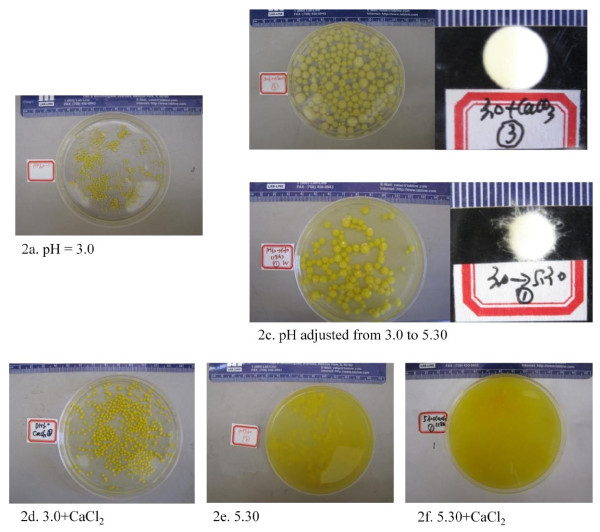
**Pelletization of *M. circinelloides *under different conditions**. **(a) **pH 3.0. **(b) **pH 3.0 + CaCO_3 _(pH changed from 3.0 to 5.30). **(c) **pH adjusted from 3.0 to 5.30. **(d) **pH 3.0 + CaCl_2_. **(e) **pH 5.30. **(f) **pH 5.30 + CaCl_2_.

Starting the cell cultures at an initial pH of 3, many very tiny, loosely packed pellets or cell aggregates could be seen in the fermentation flasks. This observation did not change even when cultivation was extended to 6 days without calcium carbonate addition, as shown in Figure 2a, where the fungal cells showed minimum growth and the broth solution remained crystal clear because of the inhibition of low pH on the cell growth of *M. circinelloides*. With the addition of calcium carbonate powder, the pH of the fermentation broth increased from 3 to 5.3, and we started to see rapid cell growth in the continued cultivation. Fungal pellets formed within around 12 hours of culture after CaCO_3 _(0.4 g/100 mL or 0.004 mol/100 mL) was added into the cell cultivation (Figure 2b). In the third group of experiments, fungal pellets formed within around 12 hours of culture after the pH of the fermentation broth was adjusted to 5.3 by adding NaOH solution at a cultivation time of 18 hours (Figure 2c) without adding CaCO_3_. In the fourth group of experiments, the fungal cells still showed limited growth after only CaCl_2 _solution (0.004 mol/100 mL) was added without changing the pH (Figure 2d). The average size of cell aggregates in this condition was doubled compared to the cell aggregates formed in the culture with no addition of CaCl_2 _or pH adjustment (Figure 2a). Low pH contributed to the small size of the cell aggregates because of limited growth and cell accumulation. No pellets were found in any of the experiments with an initial pH of 5.3, with or without addition of CaCl_2 _during cell growth (Figures 2e and 2f). No pellets were formed for cell cultures to use wood powder as nuclei without the pH shift (data not shown).

**Figure 3:**
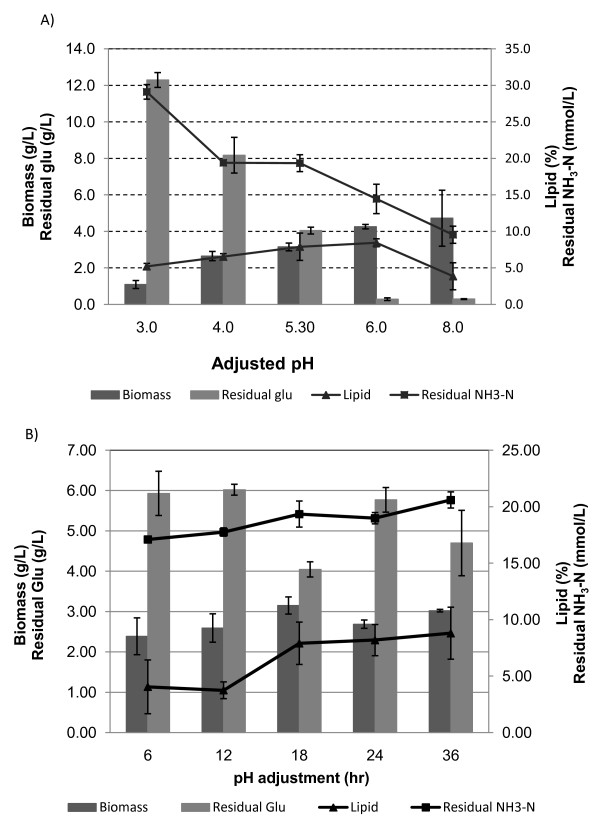
**Influence of pH adjustment during the cultivation of *M. circinelloides***. **(a) **pH adjustment to different level. **(b) **Different time to adjust pH.

The pellet formations shown in Figure 2b are white or milklike white, compact, spherical, with a smooth surface, and similar to the alginate calcium gel beads that are widely used in cell immobilization. The pellet formations shown in Figure 3c occurred with only a pH adjustment and without adding calcium carbonate. They are generally yellow, loosely packed and obviously darker than the beads shown in Figure 2b, with a fluffy surface on which some of the hyphae stretching outside can be clearly seen. In both situations, the viscosity of the cell cultivation broth was significantly lower than the cultures with a starting pH of 5.3, at which no pellets were formed (Figure 2e).

### Effects of adjustment pH on the cultivation of *M. circinelloides*

Adjusting pH during the cell cultivation was necessary for the cultivation of *M. circinelloides *because low cultivation pH inhibited both cell growth and lipid accumulation. Adding CaCO_3 _at the referenced amount (4 g/L) adjusted the pH of the fermentation broth only to 5.3 at 18 hours of cultivation. pH was adjusted to different levels by using NaOH instead of CaCO_3 _as shown in Figure 3a. This pH adjustment at 18 hours of cultivation stimulated cell growth, and higher final biomass concentration and higher glucose and ammonia consumption were recorded (Figure 3a). The pH shift started to have some negative effects once the pH was adjusted to alkaline conditions (for example, pH 8), and it caused significantly less lipid accumulation. The lipid content overall was not changed with the different level of pH adjustment, while we can see that it slightly peaked when the pH was adjusted to 6.0. All of the cell cultures with the pH adjustment at 18 hours formed pellets.

### Timing of pH adjustment during cultivation

Different times to adjust pH to 5.3 at 18 hours of cultivation overall did not show any significant effects on cell biomass and lipid accumulation (Figure 3b). By using pairwise comparison, the residual glucose concentration was relatively lower when the pH was adjusted at either 18 hours or 36 hours than at the other times tested. The lipid content of the cell biomass was higher when the pH was adjusted at 18 hours (or later) of culture than under other conditions. Adjustment of pH within 12 hours of cultivation did show some negative effects on cell pelletization, especially at a higher cultivation temperature (28°C). Only a few pellets were formed, and the majority of cell biomass remained with clumplike morphology when pH was adjusted at 12 hours. Pellets could barely be seen when pH was adjusted at 6 hours of cultivation (Table 2).

**Table 2:** Effect of different times to adjust pH on pelletization of *M. circinelloides*^a^

Different time to adjust pH from 3.00 to 5.30 (once), hours	24°C	28°C
6	○	○(few)
12	○	○(a few)
18	○	○
24	○	○
36	○	○

^a^○: pellets; ×: no pellets; ○(few): number of pellets less than 10; ○(a few): number of pellets between 10 and 30.

### Nitrogen effects on the cultivation of *M. circinelloides*

**Figure 4:**
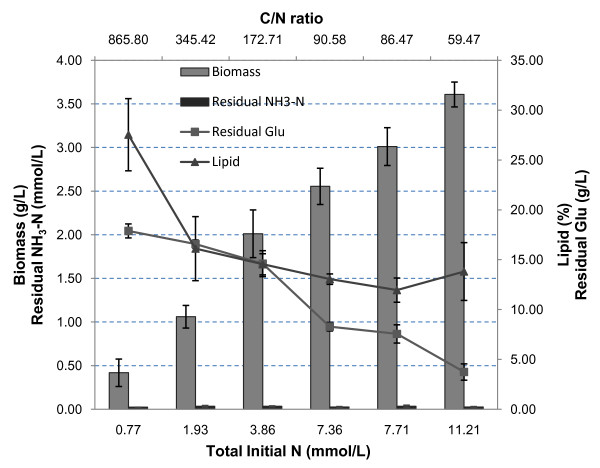
**Influence of the carbon-to-nitrogen ratio on the cultivation of *M. circinelloides***.

Similarly to its effect on many other oleaginous species, nitrogen depletion plays a key role in stimulating lipid accumulation. With the increase in the initial nitrogen level, biomass concentration increased gradually, as did glucose consumption. For all the nitrogen levels shown in Figure 4, all cultures exhibited nitrogen depletion with the final ammonia concentration dropping to around zero. At the highest carbon-to-nitrogen ratio, shown in Figure 4, the cultures had the highest lipid content, reaching almost 30% of the dry cell weight, although with relatively lower overall lipid productivity due to the limited amount of biomass that the cultures generated. Nitrogen levels did not have any effect on pelletization within this tested range, because all the cultures formed fungal pellets (Table 3). The influence of higher carbon-to-nitrogen ratios on pellet size was not significant.

**Table 3:** Effect of nitrogen concentration on pelletization of *M. circinelloides*

	Initial nitrogen concentration					
	
Parameter	0.77	1.93	3.86	7.36	7.71	11.21
Carbon-to-nitrogen ratio	865.8	345.4	172.7	90.6	86.5	59.5
Mean pellet number/100 mL (± SD)	75 ± 57	286 ± 157	376 ± 304	363 ± 39	197 ± 103	185 ± 16
Average size, mm (± SD)	1.5 ± 1.2	1.5 ± 0.8	1.9 ± 1.0	2.3 ± 1.0	3.3 ± 1.4	3.4 ± 1.3

### Temperature effects on the cultivation of *M. circinelloides*

**Figure 5:**
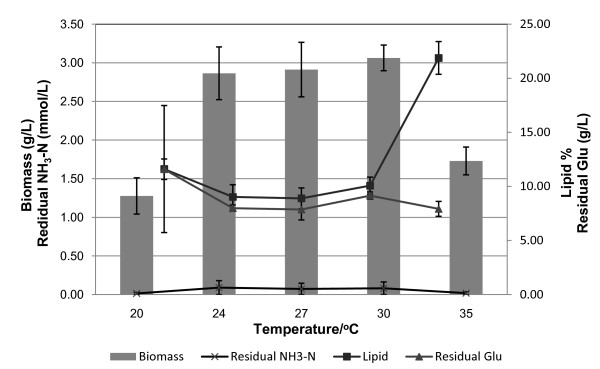
**Influence of cultivation temperatures on the culture of *M. circinelloides***.

*M. circinelloides *has the best growth temperature, ranging from 24°C to 30°C (Figure 5). Within this temperature range, the cell biomass and lipid accumulation remained almost constant, as did nitrogen and glucose consumption. A lower temperature such as 20°C significantly decreased the cell growth rate, while glucose and nitrogen consumption were not significantly different at this temperature. Similar results were found when the temperature reached 35°C. The cell biomass accumulation significantly dropped; however, the lipid content dramatically increased from about 10% to over 22%. Lipid accumulation always was triggered as long as stress conditions inhibited cell growth, and gear the available nutrient for lipid accumulation at 35°C. The cells almost stopped growing if the cultivation temperature reached 37°C, and the lipid content also significantly dropped (data not shown). Fungal cells all formed pellets with the pH shift even though we changed the culture temperature, and the number and size of the pellets formed in the flask culture were related to cell growth. The sizes of the pellets were generally smaller at 20°C because of the limited cell growth, and the number of pellets formed during the cultures at 35°C was significantly lower than that during the cultures at optimum temperatures (Table 4).

**Table 4:** Effect of culture temperature on pelletization of *M. circinelloides*

	Culture temperature				
	
Parameter	20°C	24°C	27°C	30°C	35°C
Mean pellet number/100 mL (± SD)	146 ± 102	186 ± 108	189 ± 118	197 ± 118	34 ± 22
Average size, mm (± SD)	1.4 ± 0.5	2.4 ± 1.4	2.4 ± 1.2	2.1 ± 0.5	2.6 ± 0.7

## Discussion

### Cell growth and oil accumulation

Lipid accumulation was usually triggered when cell growth was inhibited under various conditions, such as nutrient depletion and harsh environmental conditions. This was especially true for nitrogen deficiency, when *M. circinelloides *significantly increased its lipid accumulation with the lower amount of nitrogen input while the cell biomass growth was inhibited. These results confirm that filamentous fungi *M. circinelloides *were similar to other commonly used oleaginous microalgae and yeast species [28-30]. In addition to nitrogen depletion, the most commonly seen stress factor, in the present study we observed that high temperature also served as a stress factor that induced lipid synthesis. Several previous reports indicated that temperature was an important factor in regulating fatty acid composition in fungi [31,32]. Actually, lower cultivation temperature was widely applied to obtain higher production of polyunsaturated fatty acids. The effects of temperature on the total amount of lipids accumulated during the cell culture were recently studied in two microalgae strains: *Nannochloropsis oculata *and *Chlorella vulgaris*. The variation of temperature strongly influenced the lipid content of microalgae. The growth of *C*. *vulgaris *was not significantly influenced by temperature, but a decrease from 30°C to 25°C brought about lipid content that was 2.5 times higher. For *N. oculata*, elevated cultivation temperature caused increased lipid content with reduced cell growth, and lipid productivity seemed not to have changed within the range tested [33].

Similarly to oleaginous microalgae and yeast strains, the cell growth of *M. circinelloides *is fostered by niche nutritional and environmental factors such as the right carbon-to-nitrogen ratio, appropriate pH and temperature range and so on. *M. circinelloides *can tolerate much harsher environmental conditions. Although lower pH significantly inhibited its cell growth, it still demonstrated wider tolerance to different pH levels. A high carbon-to-nitrogen ratio can significantly stimulate the lipid content of *M. circinelloides *cells, which can tolerate up to 35°C. This fungus was reported to have the capability to assimilate different types of carbon sources, such as xylose, glycerol and arabinose, and to directly utilize polysaccharides, such as starch and cellulose [13,34]. Compared to other oleaginous species of industrial interest, these are tremendous features that may make lipid accumulation with the fungus more suitable in the utilization of waste materials for biofuel production, where in many cases the requirements for producing strains are much higher.

### Fungal cell pelletization

Fungal cell pelletization has been widely researched because of its several advantages, such as increasing performance on the mixing and mass transfer properties caused by viscosity, decrease of the fermentation broth, easier separation and so forth [17,19,35]. Various factors have been discovered to influence pellet formation, including culture medium, inoculum concentration, addition of nuclei and/or polymer, pH, agitation and so on [35]. Our research has primarily confirmed some of these findings. Pelletized fungal broth solution is generally not viscous and usually deviates from Newtonian behavior only at high biomass concentrations. Yet, this process is not preferred in some applications, because many metabolites produced were reduced in the pelleted form as a result of the mass transfer barrier, especially with regard to oxygen [36]. However, our results reveal that pellet formation in the cell cultivation of *M. circinelloides *did not have any negative effects on biomass and lipid accumulation (Table 1) and that sometimes pelletized fungal cultures had even better growth performance than the cakelike morphology (Figure 3b). This leads to an important conclusion that pelletized cell cultivation can be introduced into the microbial lipid accumulation process. In reality, this might potentially bring tremendous advantages to lipid and biofuel fermentation because the final cell products can be harvested by simple filtration, a much easier method than current widely used methods such as centrifugation. The addition of calcium carbonate in our research was proved to facilitate pellet formation (Figure 2b), and this approach has received wide recommendation in many recently published journal articles [35]. Calcium carbonate powder can have three effects on cell cultivation: first, it may serve as nuclei so that fungal spores can attach to its surface to develop the pellets; second, it may bring calcium ions to the fermentation broth; and third, the addition of calcium carbonate increases the pH of the fermentation broth from 3 to 5.3 as we recorded. The pH of the fermentation broth usually dropped during cell cultivation because of some unknown acid production, and pH adjustment was necessary to foster the cell growth of *M. circinelloides*. In our investigation, pH adjustment played the key role in facilitating pelletization. The cultures in which pH was adjusted only by adding NaOH caused the formation of pellets (Figure 2c), while the cultures in which only CaCl_2 _was added did not form any pellets (Figure 2f). The current terminology used to describe pellets in this field is poorly defined and subjective. Sometimes it is difficult to distinguish small cell aggregates from the pellets we usually refer to. Without adjusting pH, the fermentation solution contains many small cell aggregates, and we may argue that those are just small pellets (Figure 2a). If the latter is true, the adjustment of pH facilitated the pelletization process because it provided the right growing conditions to let the small pellets (cell aggregates) become pellets as we normally define them (Figure 2c). The influence of culture pH on fungal morphology and pelletization has been disputed in the literature [36]. One of the specific features of our pelletized fermentation process was that pH dramatically dropped during the early stage of cell cultivation, causing the growth rate to decrease considerably. This, in fact, leads to a poor cell growth phase in which only very limited cell biomass was available in the beginning phase so that only small, growth-limited agglomerates (cell aggregates or small pellets) were produced. Then, with the pH adjustment, the cultivation conditions became temperate so that hyphae quickly grew on these small agglomerates to form the pellets. This actually was not revealed in any of the research related to fungal cell pelletization, and it may provide a handy approach for the industry to use to control fungal morphology. Adjusting pH during cell cultivation is a much easier process by which to induce cell pelletization than the addition of calcium carbonate, which may drive up costs and cause solid waste disposal issues. The addition of calcium ions has more effects on the pellet structure than on the pellet formation itself. We did see that the sizes of cell aggregates (pellets) (Figure 2d) were significantly larger than the ones in which calcium ions were not added to the culture (Figure 2a). The pellets formed with the addition of calcium (Figure 2b) were much smoother on the surface than the ones formed with only pH adjustment (Figure 2c). The exact processes involved in pellet formation in fungi are not fully understood, and several mechanisms are involved in explaining the pelletization process, although they are highly strain-specific. The structure of mycelial pellets can range from loose, irregular aggregates, often with protruding hyphae (termed "fluffy" pellets), to tight, compact spheres [36]. Adding calcium ions during this process caused the process to form more compact pellets with a smooth surface. Calcium may serve as a cross-linking agency to facilitate the agglomeration of fungal cells, but the detailed effect is not clear at this stage. The nuclei effect for the calcium carbonate powder to initiate the pelletization process has not been proven, and conclusions are the same for wood powder serving as the nuclei.

## Conclusions

This study is the first to report the use of pH adjustment to induce the formation of fungal cell pelletization, and it provides a handy method by which to facilitate the cell harvest of oleaginous cells. *M. circinelloides *showed excellent performance in forming cell pellets, and its cell growth and lipid content were not significantly affected by pelletization. Similarly to other oleaginous species, lipid accumulation of *M. circinelloides *was triggered at stressed conditions such as high carbon-to-nitrogen ratio and high temperature.

## Competing interests

The authors declare that they have no competing interests.

## Authors' contributions

CX collected all experimental data, participated in the conception and design, and revised the manuscript. JZ participated in the conception and design, assisted in experiment data collection and revised the manuscript. WZ participated in conception and design. BH initiated the research idea, participated in the conception and design, data analysis and interpretation, and drafted the manuscript. All authors read and approved the final manuscript.
